# Cholesterol Depletion Regulates Axonal Growth and Enhances Central and Peripheral Nerve Regeneration

**DOI:** 10.3389/fncel.2019.00040

**Published:** 2019-02-12

**Authors:** Cristina Roselló-Busquets, Natalia de la Oliva, Ramón Martínez-Mármol, Marc Hernaiz-Llorens, Marta Pascual, Ashraf Muhaisen, Xavier Navarro, Jaume del Valle, Eduardo Soriano

**Affiliations:** ^1^Department of Cell Biology, Physiology and Immunology, Faculty of Biology, Institute of Neurosciences, University of Barcelona, Barcelona, Spain; ^2^Centro de Investigación Biomédica en Red sobre Enfermedades Neurodegenerativas (CIBERNED), Instituto de Salud Carlos III, Madrid, Spain; ^3^Department of Cell Biology, Physiology and Immunology, Institute of Neurosciences, Universitat Autònoma de Barcelona, Bellaterra, Spain; ^4^Vall d’Hebron Research Institute (VHIR), Barcelona, Spain; ^5^ICREA Academia, Barcelona, Spain

**Keywords:** regeneration, cholesterol, lipid rafts, axon growth, growth cone, filopodia

## Abstract

Axonal growth during normal development and axonal regeneration rely on the action of many receptor signaling systems and complexes, most of them located in specialized raft membrane microdomains with a precise lipid composition. Cholesterol is a component of membrane rafts and the integrity of these structures depends on the concentrations present of this compound. Here we explored the effect of cholesterol depletion in both developing neurons and regenerating axons. First, we show that cholesterol depletion *in vitro* in developing neurons from the central and peripheral nervous systems increases the size of growth cones, the density of filopodium-like structures and the number of neurite branching points. Next, we demonstrate that cholesterol depletion enhances axonal regeneration after axotomy *in vitro* both in a microfluidic system using dissociated hippocampal neurons and in a slice-coculture organotypic model of axotomy and regeneration. Finally, using axotomy experiments in the sciatic nerve, we also show that cholesterol depletion favors axonal regeneration *in vivo*. Importantly, the enhanced regeneration observed in peripheral axons also correlated with earlier electrophysiological responses, thereby indicating functional recovery following the regeneration. Taken together, our results suggest that cholesterol depletion *per se* is able to promote axonal growth in developing axons and to increase axonal regeneration *in vitro* and *in vivo* both in the central and peripheral nervous systems.

## Introduction

Axonal guidance during the development of the nervous system is thought to be highly regulated through interactions of transmembrane receptors with attractive, repulsive, and trophic cues. Similar mechanisms regulate axonal regeneration after injury. The transected axon undergoes morphological changes to form the growth cone, a highly dynamic structure that senses the environment and leads the regenerative growth (Allodi et al., 2012). Membrane receptors localized in the growth cone have an important role in axonal signaling (Guirland et al., 2004; Kamiguchi, 2006). The regenerative shift of axotomized neurons is promoted by injury-induced signals, which stimulate the transcription of various trophic factors, adhesion molecules, growth-associated proteins and structural components needed for axonal regrowth and cell survival (Rishal and Fainzilber, 2014).

Many intrinsic and extrinsic signals can promote or inhibit axonal regeneration. Due to the importance of these signals during axonal degeneration and regeneration after peripheral nerve injury, microdomains in the membrane that cluster a range of proteins and molecules related to cellular signaling may play a key role in the regulation of these pathways. In particular, lipid rafts have been described as cholesterol-enriched cell membrane microdomains that compartmentalize lipids and protein to form signaling platforms (Golub et al., 2004).

Cholesterol is a major component of the nervous system, being essential for normal brain development. The brain is the most cholesterol-rich organ, containing about 20% of the body’s total cholesterol (Bjorkhem and Meaney, 2004). Under normal physiological situations and because plasma lipoproteins do not cross the intact blood-brain barrier, nearly all cholesterol in the brain is synthesized *in situ* (Dietschy and Turley, 2001). Brain cholesterol is an important structural component of cellular membranes and myelin. It is also required for the synthesis of steroid hormones and for the organization of lipid rafts, which are involved in many aspects of brain function, such as growth factor signaling, synapse and dendritic formation (Goritz et al., 2005), and axon elongation and guidance (de Chaves et al., 1997).

Here we studied the effects of altered membrane integrity by reducing the cholesterol content in the axons of three neuronal systems, namely hippocampal and cerebellar external granular layer (EGL) cells as a Central Nervous System (CNS) example, and the dorsal root ganglion (DRG) neurons as a Peripheral Nervous System (PNS) example. We show that depletion of cholesterol leads to increased sizes of growth cones, filopodial extensions and neurite length. Moreover, we also demonstrate that cholesterol membrane and raft disruption increase the regenerative capacity of axons after axotomy both *in vitro* and *in vivo* and enhance muscle and sensory re-innervation of distal targets. On the basis of our findings, we propose that acute reduction of neuronal cholesterol emerges as a potential therapeutic strategy to improve regenerative outcomes after peripheral nerve lesion.

## Materials and Methods

### Reagents and Antibodies

The following antibodies were used: anti-GFP (A11122, Invitrogen); anti-IIIβ-tubulin (MMS-435P, Covance); anti-growth associated protein 43 (GAP43) (AB5220, Millipore); anti-myelin basic protein antibody (MBP) (ab7349, Abcam); anti-neurofilament H (NF-H) (AB5539, Millipore); Donkey anti-Mouse IgG (H+L) Highly Cross-Adsorbed Secondary Antibody Alexa Fluor 488 (A-21202, Thermo Fisher); Donkey anti-Rabbit IgG (H+L) Highly Cross-Adsorbed Secondary Antibody Alexa Fluor 488 (A-21206, Thermo Fisher); Goat anti-Chicken IgY (H+L) Alexa Fluor 488 (A-11039, Thermo Fisher), Biotinylated Horse anti-rabbit IgG (BA-1000, Vector); Biotinylated Goat anti-rat IgG (BA-9400, Vector), Streptavidin-Biotinylated HRP Complex (RPN1051, GE Healthcare); and Streptavidin-Alexa Fluor 594 (S32356, Thermo Fisher).

The following drugs and reagents were used: poly-D-Lysine (P7280, Sigma); laminin (L2020, Sigma); Nystatin dihydrate (N4014, Sigma); Cholesterol Oxidase *Streptomyces* sp. (ChOx) (228250, Calbiochem); Methyl-β-cyclodextrin (MβCD) (C4555, Sigma); DMSO (D5879, Sigma); phalloidin – TRITC (P1951, Sigma); biocytin (B4261, Sigma); Cholera Toxin Subunit B (Recombinant) Alexa Fluor 594 (CTxB-594) (C34777, Life BioSciences).

### Primary Cultures

#### Hippocampus

Primary cultures of mouse hippocampi were prepared from E16-E17. Pregnant CD1mice were sacrificed by cervical dislocation, and the fetuses were collected in a PBS-glucose 0.3% solution and then decapitated. Hippocampi were isolated and trypsinized for 6 min at 37°C. Trypsin was then neutralized with FBS and incubated with DNase for 10 min at 37°C. Neurons were then centrifuged at 800 rpm for 5 min, resuspended and plated in pre-coated culture glasses with poly-D-lysine in medium containing Neurobasal (w/o L-glutamine, w/Phenol Red; GIBCO, 21103-049), 1% penicillin/streptomycin (GIBCO, 15140-122), 1% glutamine (GIBCO, 25030-024) and 2% B27 (GIBCO, 17504-044).

#### Cerebellum

Primary cultures of cerebellums were prepared from P4–P5 CD1 mice sacrificed by decapitation. Cerebellums were isolated, mechanically disaggregated and trypsinized as previously described. After centrifugation, neurons were resuspended in 2 mL of DMEM, and EGL were isolated by centrifugation in a percol gradient. After a wash centrifugation, EGL were plated in pre-coated culture glasses with poly-D-lysine in medium containing DMEM (GIBCO, 41966-029), 1% penicillin/streptomycin (GIBCO, 15140-122), 1% glutamine (GIBCO, 25030-024), 4.5% D-(+)-Glucose (Sigma, G-8769), 5% NHS (GIBCO, 26050-088), and 10% FBS (GIBCO, 16000-044) for 24 h, and then NHS and FBS were replaced by 2% B27 (GIBCO, 17504-044) and 1% N2 (GIBCO, 17502-048).

#### Dorsal Root Ganglion (DRG)

Primary cultures of DRG neurons were prepared from E13–E14 mice. Pregnant CD1 mice were sacrificed by cervical dislocation, and the fetuses were collected and decapitated. DRG neurons were isolated and trypsinized as previously described. After centrifugation, neurons were resuspended and plated in pre-coated culture glasses with poly-D-lysine and laminin in medium containing DMEM (GIBCO, 41966-029), 1% penicillin/streptomycin (GIBCO, 15140-122), 1% glutamine (GIBCO, 25030-024), 0.06% D-(+)-Glucose (Sigma, G-8769), 0.0045% NaHCO3 (GIBCO, 25080-060), 2% B27 (GIBCO, 17504-044) and 5 μg/ml NGF.

Organotypic entorhino-hippocampal slice cultures were obtained from P0 actin-GFP and WT mice sacrificed by decapitation. Horizontal sections 325 μm thick containing both the entorhinal cortex (EC) and the hippocampus were obtained by cutting tissue pieces in a chopper (McIlwain Tissue Chopper, Standard Table, 220V, Prod 10180-220). Slices were laid on a porous Millicell CM plate culture insert (Millipore, PICM03050) and incubated using the interface culture technique. The medium comprised 35.5% Neurobasal, 25% MEM powder, 25% NHS (GIBCO, 26050-088), 12.5%HBSS (w/Mg, w/Phenol Red, GIBCO, 24020-083), 0.5% D-(+)-glucose (Sigma, G-8769), 1% glutamine (GIBCO, 25030-024), 1% penicillin/streptomycin (GIBCO, 15140-122), 1.1% sodium pyruvate (Sigma, P2256-5G), 0.04% NaHCO3 (GIBCO, 25080-060), 2% B27 (GIBCO, 17504-044) and 1% N2 (GIBCO, 17502-048). The medium was changed every 2 days. After 21 days *in vitro* (DIV), axotomy was performed between the EC and the hippocampus with a needle, and WT hippocampi were put with EC GFP. The cultures were treated for 10 days with Nystatin or control medium and fixed with 4% paraformaldehyde (PFA) for 1h. Cultures were cut into 60 μm slices with a vibratome (Leica VT 1000 S) and cryopreserved at -20°C until immunohistochemistry was performed.

### Drug Treatments *in vitro*

#### Growth Cone And Filopodium Experiments

Hippocampal and EGL primary cultures were treated after 3 DIV and DRG after 1 DIV with 2.5 μg/mL Nystatin for 10 min, 0.5 mM MβCD for 10 min or 2U ChOx for 2 h. They were then incubated for an additional 30 min in culture media (Neurobasal or DMEM) and then fixed in 4% PFA.

#### Axon Extension And Branching Experiments in DRG Neurons

After 2 h in culture, 2.5 μg/mL Nystatin and 2U ChOx were added to the media for 24 h and neurons were then fixed. After 22 h *in vitro*, and therefore 2 h before neurons were fixed, 0.5 mM MβCD was added to the media.

#### Axotomy Experiments

Axotomy in microfluidic chambers (AX50010TC, Millipore) was performed with a pipette connected to a vacuum pump. Complete axotomy was verified in the inverted microscope. Immediately after axotomy, 2.5 μg/mL Nystatin was added and maintained in the culture for 3 days. In organotypic cultures, 5 μg/mL Nystatin was added to the media just after axotomy and maintained for 10 days, changing the media every 2 days.

### Animals and Surgical Procedures

To study the effect of cholesterol depletion on healthy mice, 10 OF1 female mice (20–25 g) were divided in two groups and treated with saline (vehicle) or MβCD 1000 mg/kg/week intraperitoneal (i.p.) during 1 month. To study the effect of lipid raft disruption on peripheral nerve regeneration, 13 female mice (25–30 g) were injured on the sciatic nerve and allowed to regenerate for 1 month while receiving saline (*n* = 6) or 1000 mg/kg/week MβCD (*n* = 7) i.p. treatment.

To perform the nerve injury, animals were anesthetized by i.p. injection of ketamine (90 mg/kg; Imalgene 500, Rhône-Merieux, Lyon, France) and xylazine (10 mg/kg; Rompun, Bayer, Leverkusen, Germany). The right sciatic nerve was surgically exposed at the midthigh and carefully freed from adherences to surrounding tissues. Afterwards, it was cut at 45 mm from the tip of the 3rd toe and immediately repaired using two epineurial sutures (10–0), maintaining the fascicular alignment of the sciatic branches. Finally, the wound was sutured in planes and disinfected. Animals were left to recover on a hot pad after being returned to their cages. The left paw was left uninjured as a control.

All the animals had food and water *ad libitum* and were kept at a standard temperature (22 ± 2°C) and under 12:12-h light-dark cycles (300 lux/0 lux). The experimental procedures followed the recommendations of the European Communities Council Directive 2010/63/EU for the care and use of laboratory animals and were approved by the Committee for Ethics on Experimental Animal and Human Research of the Universitat Autònoma de Barcelona.

### Electrophysiology Tests

To test possible effects of MβCD on intact animals, electrophysiological tests were performed every 7 days after beginning of the treatment and for 4 weeks. To monitor peripheral nerve regeneration, electrophysiological tests were performed at 14, 21, 25, 28, and 33 days post-injury (dpi).

Animals were anesthetized with pentobarbital (10 mg/kg) and the sciatic nerve was stimulated using two needle electrodes inserted percutaneously at the sciatic notch, applying single rectangular pulses of 0.02 ms up to the voltage required to obtain a maximal evoked response. The compound muscle action potentials (CMAP, M wave) evoked by stimulation of motor nerve fibers were recorded from the tibialis anterior (TA) and plantar (PL) muscles with microneedle electrodes. All potentials were amplified and displayed on a digital oscilloscope (Tektronix 450S; Tektronix, Beaverton, OR, United States) at the appropriate settings for latency and amplitude measurements from the baseline to the maximal negative peak. During the tests, the animals were placed over a warmed flat coil controlled by a hot water circulating pump to maintain body skin temperature.

### Locomotion Evaluation

The DigiGait system (Mouse Specifics, Boston, MA, United States) was used to assess locomotor performance of healthy animals treated with MβCD or vehicle at weekly intervals during treatment. The mice were forced to run over the transparent belt of a motorized treadmill and recorded with a high-speed video camera (80 frames/s) from below while running at a constant treadmill velocity of 20 cm/s. A minimum of 200 images were collected for each walking mouse containing more than eight strides for each run and each video was analyzed with the DigiGait software. The print length, and the toe spread distance between toes 1–5 and toes 2–4 were measured, and the sciatic functional index (SFI) between the right and the left paw was calculated (Navarro, 2016).

### Mechanical Algesimetry

The algesimetry tests for mechanical stimuli were performed on both hindpaws every week along treatment in uninjured animals. Sensibility to a non-noxious mechanical stimulus was measured by using an electronic Von Frey algesimeter (Bioseb, Chaville, France). Mice were placed on a wire net platform in plastic chambers 10 min before the experiment for habituation. The hindpaw plantar surface was stimulated from the bottom of the box by applying a 0.4 mm non-noxious pointed metal probe to the central area of the paw, and then slowly increasing the pressure until the mouse raised the paw, with a 35 g cut off force to avoid skin damage. The mechanical nociceptive threshold was taken as the mean of three measurements per paw and the threshold was expressed as the force (in grams) at which mice withdrew the paw in response to the stimulus (Cobianchi et al., 2014).

### Pinprick Test

Progression of nociceptive reinnervation of the hindpaw was assessed at 14, 21, 25, 28, and 33 dpi by means of the pinprick test. Awake animals were gently wrapped in a cloth with the injured paw facing upward. The skin surface near C plantar pad and 4th toe (more distal) were stimulated with a blunt needle with enough intensity to indent the skin without damaging it (Cobianchi et al., 2014). Each site was stimulated twice and responses were recorded as positive only when clear pain reactions such as fast withdrawal or vocalization were triggered. Positive responses were taken as a sign of functional reinnervation of the skin.

### Histology, Immunohistochemistry and Immunocytochemistry

Neurons were fixed with a solution of 4% PFA in PBS for 10 min. Subsequently, they were rinsed with PBS and permeabilized with a solution of triton 0.1% X-100 in PBS for 10 min. Afterwards, they were rinsed with PBS once more, and blocking solution (NHS 10% in PBS) was added for 1 h at room temperature. After blocking, the cells were incubated for 2 h with the respective primary antibodies diluted in blocking solution. Unbound primary antibodies were washed with PBS, and neurons were incubated with the respective secondary antibodies in blocking solution for 1 h at room temperature. For F-actin staining after permeabilization, phalloidin diluted in PBS was added and maintained for 30 min. Neurons were mounted using Mowiol.

Organotypic slices were rinsed with PBS with 0.5% Triton three times, and were incubated for 2 h at room temperature with blocking solution (10% NHS, 0.2M Glycine in PBS-Gelatin with 0.5%Triton). After blocking, the cells were incubated overnight at 4°C with the respective primary antibodies diluted in antibody solution (5% NHS in PBS-Gelatin with 0.5%Triton). Unbound primary antibodies were washed with PBS with 0.5% Triton, and slices were incubated with the respective secondary antibodies in antibody solution for 2 h at room temperature.

For the *in vivo* regeneration experiment, mice were transcardially perfused with 4% PFA in PBS (0.1 M, pH 7.4) 1 month after beginning the treatment. Subsequently, the sciatic nerve, spinal cord, L4 and L5 DRG neurons were removed, postfixed for 24 h with PFA and cryopreserved in PB containing 30% of sucrose for immunohistochemistry analysis. Samples were serially cut (15 μm) with a cryotome (Leica CM190) and stored at -20°C for further analysis.

For cholesterol depletion analysis, samples were defrosted for 30 min at room temperature and then rehydrated with PBS for 5 min. DRG neurons were then incubated for 3 h at room temperature with CTxB (1:200) in PBS. Slides were washed and mounted using Mowiol.

For immunohistochemistry, samples were defrosted at room temperature, blocked with PBS-specific animal serum, and incubated overnight at 4°C with the primary antibodies rabbit anti- GAP43 (1:100), rat anti- MBP (1:200) and chicken anti-NF-H (1:100). After washes, sections were incubated with the respective biotinylated secondary antibody (1:200) for GAP43 and MBP and then for 1 h at room temperature with conjugated Alexa Fluor streptavidin (1:200) or Alexa Fluor anti-chicken IgY. Finally, sections were mounted with Mowiol containing DAPI for nuclear staining.

### Image Analysis

Images of primary cell cultures were taken in an epifluorescence microscope (Eclipse Nikon E1000) with a 60× oil-immersion objective. To take images from the *in vitro* axotomy experiments, we used an inverted microscope (Olympus ScanR) with a 20× objective. For images of the organotypic cultures, we used a confocal microscope (Leica TCS SP5) with a 20× objective and a 20× objective with 2× zoom to quantify axon regeneration. Images of the tibial nerve with MBP and NF-H immunostaining were taken10 mm distally from the injury site with an epifluorescence microscope (Eclipse Ni, Nikon) attached to a digital camera (DS-Ri2, Nikon). Finally, we used a confocal fluorescent microscope (Zeiss LSM 700) to acquire the images from the SC and DRG tissue sections stained with GAP43, and images were analyzed with ImageJ (Schneider et al., 2012). Briefly, the area of the soma of at least 100 DRG neurons/animal and 10 motoneurons/animal were taken as ROI, and the integrated density (mean gray value × area) was obtained. The mean value obtained for each animal was used for comparison (Romeo-Guitart et al., 2018).

### Statistical Analysis

All *in vitro* experiments were done three times independently. Data show means ± SEM. To analyze the statistics, we used GraphPad software and applied ANOVA test for multiple conditions or the Student’s *t*-test for two conditions. *In vivo* results are presented as mean ± SEM and means were compared with two-tailed, unpaired Student’s *t*-test, two-way ANOVA followed by Tukey’s Multiple Comparison Test or Mantel–Cox test. Differences were considered significant at *p* < 0.05.

## Results

### Cholesterol Depletion Increases Growth Cone Area and Number of Filopodia in Hippocampal and EGL Cerebellar Axons

**FIGURE 1:**
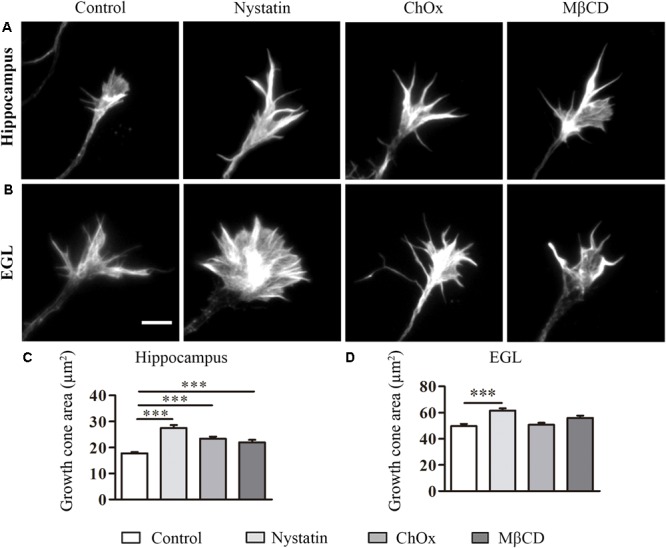
Cholesterol depletion increases growth cone area in hippocampal neurons and EGL neurons. Examples of hippocampal **(A)** and EGL **(B)** growth cones treated with control culture media, 2.5 μg/ml Nystatin, 2U ChOx, and 0.5 mM MβCD and stained with phalloidin. Growth cones areas from three independent experiments were measured. Data represent means ± SEM **(C,D)**. *N* = 150–250 growth cones in each condition (One-way ANOVA, Dunnett’s Multiple comparison test ^∗∗∗^*p* < 0.0001). Scale bar 5 μm.

Growth cones play a key role during axonal growth and guidance. First, we examined the effect of cholesterol depletion on the morphological features of growth cones. To this end, we treated hippocampal and EGL neurons with two drugs that efficiently reduce cholesterol in the cell membrane (Nystatin and MβCD), and with ChOx, a cholesterol-depleting enzyme (Figure 1) (Bolard, 1986; Cahuzac et al., 2006; Zidovetzki and Levitan, 2007). Both neuron types were kept in culture for 3 DIV before being treated with the aforementioned drugs. In hippocampal neurons, depletion of cholesterol driven by the three drugs significantly increased the growth cone area identified by phalloidin staining (Figure 1A,C). However, only Nystatin increased this parameter in EGL neurons (Figure 1B,D). Overall, our results show that cholesterol depletion increases the growth cone area in hippocampal and EGL neurons in the CNS.

**FIGURE 2:**
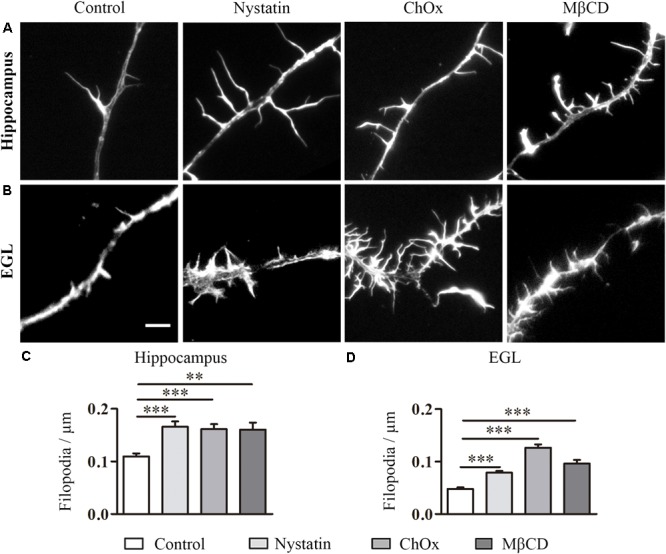
Cholesterol depletion increases filopodium-like structures in hippocampal neurons and EGL neurons. Examples of hippocampal **(A)** and EGL **(B)** filopodium-like structures in axons treated with control culture media, 2.5 μg/ml Nystatin, 2U ChOx, and 0.5 mM MβCD and stained with phalloidin. The density of filopodium-like structures was measured in discrete segments of axons in three independent experiments. Data represent means ± SEM **(C,D)**. *N* = 150–250 neurons in each condition (One-way ANOVA, Dunnett’s Multiple comparison test ^∗∗^*p* < 0.01, ^∗∗∗^*p* < 0.0001). Scale bar 5 μm.

During the course of the experiments, we observed another effect of cholesterol depletion, namely the development of numerous filpodium-like extensions/branching points along neurites. Filopodia, dynamic membranous structures driven from the actin cytoskeleton, are necessary for axonal motility, guidance, branching and regeneration. To determine the importance of cholesterol in the formation of filopodium-like structures, we again treated hippocampal and EGL neurons with Nystatin, ChOx, and MβCD (Figure 2). The filopodium-like structures were counted in discrete segments of hippocampal and EGL axons (Figure 2A,B). Cholesterol depletion induced by these drugs led to a significant increase in the number of filopodium-like structures in both types of neuron (Figure 2C,D).

These results indicate that a reduction in the cholesterol content of the cell membrane of both types of neuron promoted an increase in the number of filopodium-like structures and branching. In contrast to the effect of these drugs on growth cone area, these compounds enhanced the number of filopodia/branches in hippocampal and EGL neurons.

### Cholesterol Depletion Affects Growth Cone Area, Number of Filopodia, and Neurite Extension in DRG Neurons

**FIGURE 3:**
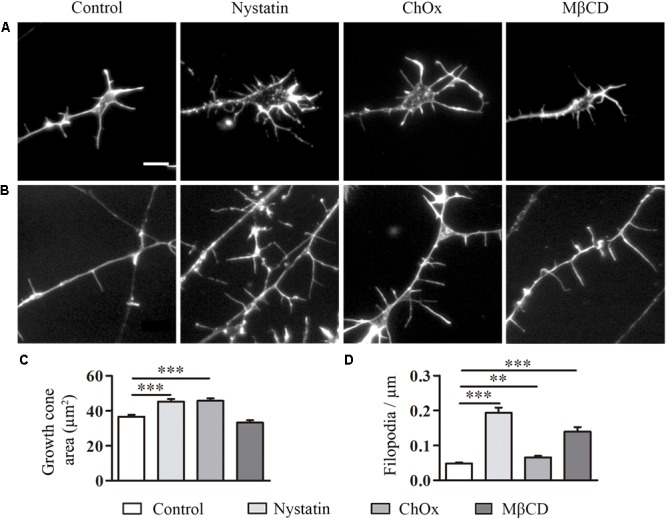
Cholesterol depletion affects growth cone area and the density of filopodia in DRG neurons. Examples of DRG growth cones **(A)** and filopodia **(B)** incubated with control media, 2.5 μg/ml Nystatin, 2U ChOx, and 0.5 mM MβCD and stained with phalloidin. Growth cone areas and filopodia from three independent experiments were measured. Data represent means ± SEM **(C,D)**. *N* = 150–250 neurons in each condition (One-way ANOVA, Dunnett’s Multiple comparison test ^∗∗^*p* < 0.01, ^∗∗∗^*p* < 0.0001). Scale bar 5 μm.

To determine whether the effects of cholesterol depletion observed in the CNS neurons can be extrapolated to the PNS, we performed the same experiments using DRG neurons (Figure 3). DRG neurons were isolated from mouse embryos at E13–14 and treated after 1 DIV with cholesterol removal agents. Treatment with Nystatin and ChOx led to an increase in growth cone area in DRG neurons (Figure 3A,C), and the removal of cholesterol with any of the three drugs enhanced the number of filopodia (Figure 3B,D).

**FIGURE 4:**
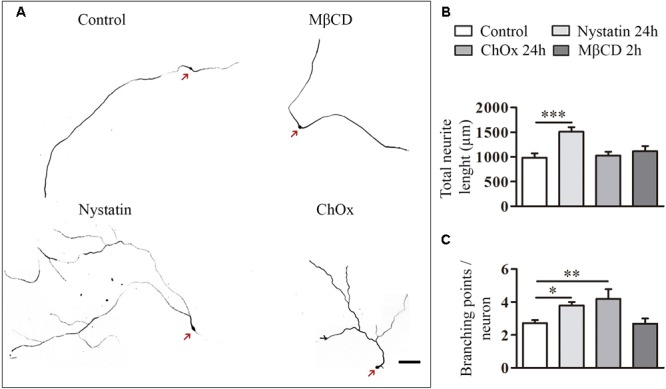
Cholesterol depletion increases neurite elongation and branching in DRG neurons. Examples of DRG neurons incubated for long periods with control media, 2.5 μg/ml Nystatin, 2U ChOx, and 0.5 mM MβCD **(A)** and immunolabeled with anti-βIII-tubulin. Total neurite extension and number of branching points in each neuron were quantified in each condition in three independent experiments. Data represent means ± SEM **(B,C)**. *N* = 30–60 neurons in each condition (One-way ANOVA, Dunnett’s Multiple comparison test ^∗^*p* < 0.05, ^∗∗^*p* < 0.01, ^∗∗∗^*p* < 0.0001). Arrows point cell body. Scale bar 100 μm.

We also quantified the effect of cholesterol depletion on neurite extension and branching (Figure 4). Treatments were performed for 24 h in the case of Nystatin and ChOx and for 2 h before fixation in the case of MβCD, as we observed increased cell death with longer incubations with this drug. Only the treatment with Nystatin increased the total length of DRG neurites while ChOx and MβCD did not affect this parameter (Figure 4A,B). We found that 24 h treatments with Nystatin and ChOx led to a significant increase in the number of branching points (Figure 4A,C).

These results suggest that pharmacological removal of cholesterol from the cell membrane has similar effects on CNS (hippocampal and cerebellar) and PNS (DRG) neurons. An acute decrease in cholesterol levels *per se* increases growth cone area and filopodia/branching point density in developing neurons.

### Cholesterol Depletion Improves Axonal Regeneration After Axotomy *in vitro* and *ex vivo*

One of the hallmarks of CNS regeneration is the difficulty to achieve the regrowth of damaged axons. This difficulty is attributed mainly to the generation of an axon blocking “milieu.” As we have shown that cholesterol depletion increases axonal growth cone area and axonal extension, we next addressed, using an *in vitro* and an *ex vivo* system, whether depletion of this lipid enhances axonal regeneration.

**FIGURE 5:**
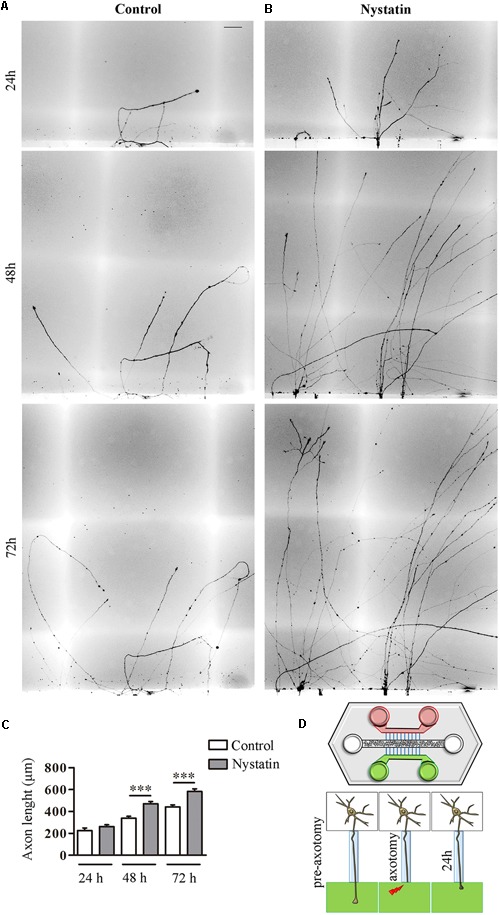
Nystatin improves axon regeneration after axotomy *in vitro*. Examples of hippocampal axon regrowth at 24, 48, and 72 h after axotomy in control condition **(A)** and in response to treatment with 2.5 μg/ml Nystatin **(B)**. Axon lengths were measured in each condition. Data represent means ± SEM **(C)**. Schematic diagram of a microfluidic chamber **(D)**. Cell bodies are in the central channel (white), axon growth through microfluidic channels (blue) to both sides (green and red). After axotomy, control medium was applied on one side (red) and Nystatin on the other (green). *N* = 3 chambers (*t*-Test in each time point, ^∗∗∗^*p* < 0.0001). Scale bar 50 μm.

First, E16 hippocampal neurons were seeded in a microfluidic chamber (Figure 5) and neurons were axotomized. This chamber allowed us to differentially treat the axons, on one side with control medium and on the other with medium supplemented with Nystatin applied immediately after axotomy (Figure 5D). Axons treated with Nystatin showed a significant increase in length when compared with those cultured in basal control medium (Figure 5A–C).

**FIGURE 6:**
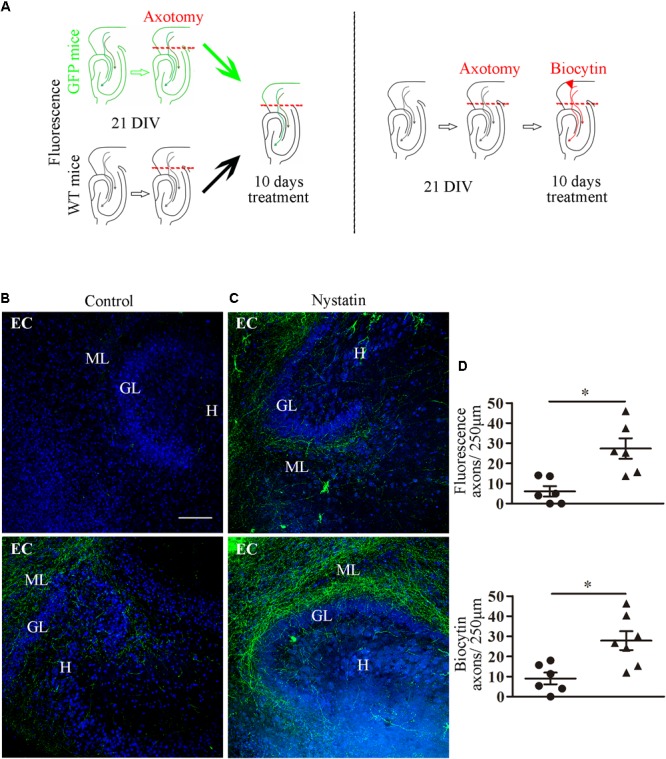
Nystatin increases EC axon regeneration in organotypic cultures. **(A)** Schematic diagram that summarizes the experimental protocol. Slice co-cultures from P0 actin-GFP and WT mice were isolated, as described in Section “Materials and Methods” and kept in culture 21 DIV. Axotomy of the EHP was performed, and slices were treated for 10 DIV with control medium or 5 μg/ml Nystatin. **(B)** Representative examples of control hippocampal slices show virtually no regenerative axons (upper panel) or very few regenerative axons (lower panel). **(C)** Examples of hippocampal slices treated with Nystatin show moderate axonal regeneration (upper panel) or robust axonal regeneration (lower panel). Axons that cross a 250 μm line were counted in three random fields near the dentate gyrus in each slice. Data represent means ± SEM **(D)**. One dot corresponds to one slice (*t*-Test ^∗^*p* < 0.05). Scale bar 100 μm. ML, molecular layer; GL, granule cell layer; H, hilus.

These results suggest that the removal of cholesterol by means of Nystatin enhances axon regeneration after axotomy *in vitro*. We next used the *ex vivo* organotypic model of axotomy and regeneration (del Rio and Soriano, 2010). Slice co-cultures from P0 mouse hippocampi and the EC were isolated and kept in culture for 21 days (Figure 6). Some EC slices were obtained from actin-GFP transgenic mice. At 21 DIV, axotomy was performed sectioning the entorhino-hippocampal connection (EHP) using a needle, and mixed co-cultures were generated. The hippocampi from actin-GFP co-cultures were removed and replaced by hippocampi from WT mice organotypic cultures, thereby allowing the direct visualization of regenerating GFP-positive EC-hippocampal axons. Axotomized organotypic cocultures were treated with Nystatin for 10 additional days (Figure 6A). In axotomized co-cultures that were not treated with Nystatin, virtually no regenerating axons (or very few) were found in the SLM/ML layers, which are the layers of termination of the EHP pathway (Figure 6B,D). In contrast, after Nystatin treatment, we observed a 10-fold increase in the density of regenerating EC axons present in the SLM/ML layers (Figure 6C,D). Organotypic cultures treated with Nystatin showed a normal morphology and distribution, and no evidence of neuronal degeneration was appreciated (Figure 6C). In another set of experiments, WT EC slice-cocultures were generated and axotomized as described above (Figure 6A) and regenerating EC axons were visualized by Biocytin injections (del Rio and Soriano, 2010). Again, the number of regenerating EHP axons was markedly increased by the incubation with Nystatin. Together with the above results, the present findings indicate that cholesterol depletion favors axonal regeneration *in vitro* and *ex vivo*.

### Treatment With MβCD Has No Effect on Healthy Mice

The results of motor electrophysiological tests performed showed that MβCD administration *in vivo* did not cause any impairment in the mice. The M wave amplitude (Supplementary Figures S1A,B) and latency (Supplementary Figures S1C,D) of TA and PL muscles did not present significant differences between MβCD treated and saline control groups. The walking track test yielded no differences in the SFI between groups (Supplementary Figure S1E). Finally, mechanical algesimetry was performed to evaluate possible changes in nociception in MβCD treated animals. Results showed no significant differences between control and treated animals in pain threshold along time (Supplementary Figure S1F).

### Treatment With MβCD Alters the Integrity of the Lipid Raft by Reducing the Amount of Membrane Cholesterol

**FIGURE 7:**
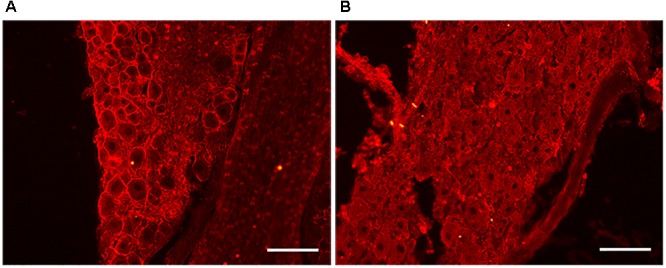
MβCD disruption of lipid rafts in DRG neurons. **(A,B)** Representative images of the DRGs of animals treated with vehicle **(A)** and MβCD **(B)**. Note the absence of CTxB membrane staining in B after MβCD treatment, indicating lipid raft disruption. Scale bar: 100 μm.

Our previous results suggest that cholesterol depletion is directly involved in promoting axonal regeneration. However, we next sought to determine the effect of acute reduction of cholesterol levels in the *in vivo* model of peripheral nerve lesion and regeneration. Cholesterol is a major component of lipid rafts, which are specialized membrane microdomains that hold a multitude of signaling receptors. It has recently been shown that disruption of the raft by acute depletion of cholesterol promotes regeneration and functional recovery after spinal cord injury (Tassew et al., 2014). Thus, we first examined the integrity of raft structures in DRG nerves after acute depletion of cholesterol by treatment with MβCD. Contralateral DRG neurons of injured mice treated with MβCD or saline were incubated with the CTxB. This fraction of the toxin has a known affinity for ganglioside GM1, a lipid raft-associated molecule (Schnaar et al., 2014) widely used as a marker of the presence of cholesterol in the cellular membrane and of lipid raft integrity. Fluorescent staining showed clear CTxB labeling in the plasma membrane of DRG neurons from animals that received vehicle for 1 month (Figure 7A) in comparison with those treated with MβCD (Figure 7B).

### Cholesterol Depletion Induced by MβCD Increases GAP43 in Sensory Neurons After Axotomy

**FIGURE 8:**
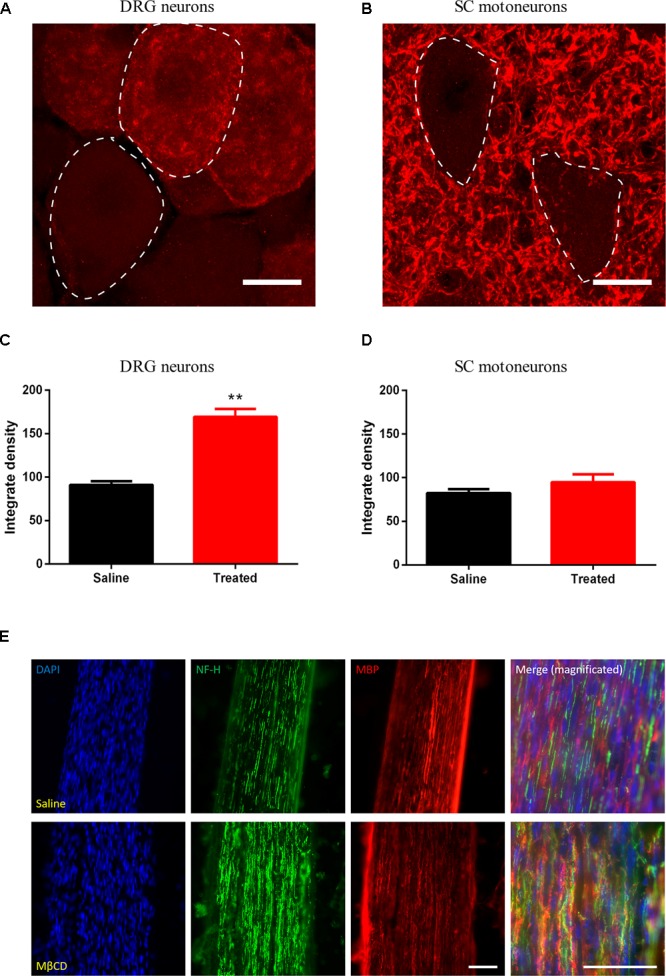
MβCD increases GAP43 expression in injured DRG neurons. **(A,B)** Detail of DRG (**A**, dashed line) and motoneurons (**B**, dashed line) analyzed for GAP43 expression. **(C,D)** Quantification of GAP43 positivity in DRG neurons **(C)** and motoneurons **(D)** from animals treated with vehicle or MβCD vs. Saline. **(E)** Detail of a regenerated nerve stained for cell nuclei (blue), NF-H (green), and GAP43 (red) of saline- (top) and MβCD-treated (bottom) animals. Scale bar: 15 μm in **(A,B)**; 50 μm in **(E)**. Student’s *t*-test, ^∗∗^*p* < 0.01.

To study the regenerative response in sensory (Figure 8A) and motor (Figure 8B) neurons after nerve section, the expression of the GAP43 was measured. GAP43 is expressed at high levels during development and axonal regeneration, and it is considered a crucial component of an effective regenerative response in the nervous system, being used as a marker of regeneration in injured axons. The results showed higher immunoreactivity for GAP43 in DRG neurons in injured animals treated with MβCD compared to those treated with vehicle (Figure 8C). This observation indicates that cholesterol depletion after sciatic injury enhances the neuronal regenerative program. No significant difference was observed for motoneurons (Figure 8D). Moreover, myelin staining of the regenerated nerve was conducted to assess whether lipid raft disruption could affect axonal remyelination. Qualitative assessment of both saline- and MβCD-treated animals (Figure 8E) shows similar presence of myelin in nerve fibers of both groups, thus indicating that lipid raft disruption did not block remyelination after injury.

### Cholesterol Depletion Induced by MβCD Improves Muscle Reinnervation and Sensory Responses in Mice

**FIGURE 9:**
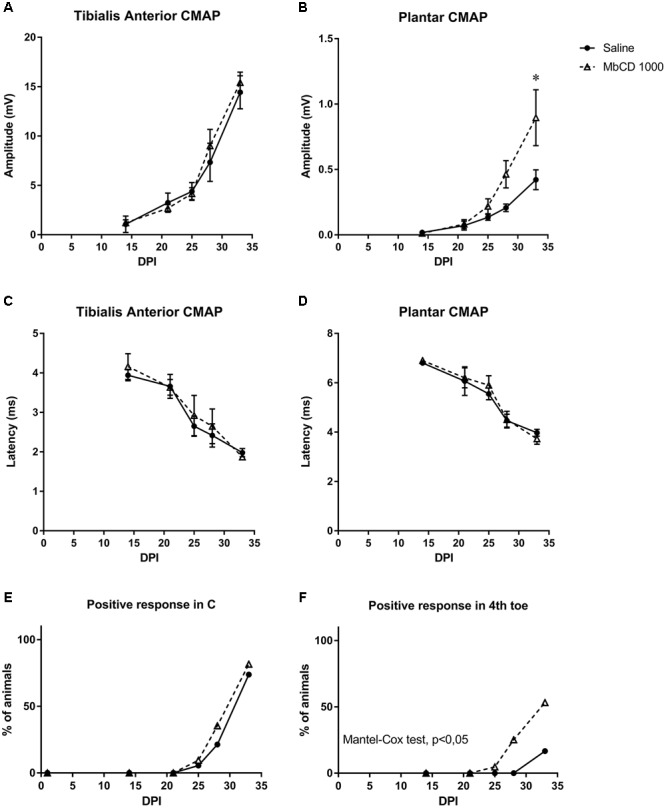
MβCD treatment accelerates motor and sensory functional recovery following sciatic nerve injury. **(A–D)** Electrophysiological results after nerve injury in mice treated with vehicle or with MβCD. Results of CMAP amplitude **(A,B)** and latency **(C,D)** recorded in TA and PL at 14, 21, 25, 28, and 33 dpi. Data are shown as the mean and SEM (bars). ^∗^*p* < 0.05 with two-way Anova and Bonferroni test. **(E,F)** Pinprick test results to analyze sensory reinnervation in the hindpaw pad C **(E)** and 4th toe **(F)**. Results show the percentage of animals with positive response in each area in the groups treated with vehicle or MβCD. *p* < 0.05 Mantel–Cox test indicate differences in the proportion of mice with reinnervation.

Electrophysiological tests performed to follow up nerve regeneration after sciatic nerve section and repair showed a progressive increase in the amplitude of the M wave of the two muscles in both groups, thereby indicating progressive reinnervation of the muscles by the regenerated axons. Although no differences were seen for the proximal TA muscle, MβCD-treated mice had significantly higher M wave amplitude of the PL muscle than control animals at 33 days (Figure 9A,B), indicative of greater reinnervation of the distal muscles. Values of M wave latency did not show significant differences between the two groups (Figure 9C,D).

No signs of autotomy, that could be indicative of neuropathic pain, were found in any studied mice. Recovery of sensitivity was studied by means of the pinprick test on areas of the paw from proximal to distal region of the sciatic nerve innervation territory (Cobianchi et al., 2014). In the C plantar pad, no animals showed positive response at 21 days, and with time more animals recovered sensory response without differences between groups. However, in the 4th toe, which is the most distal area, cholesterol-depleted mice showed positive response earlier than injured controls, and at 33 dpi, 50% of the MβCD group showed positive responses whilst only 12.5% of the vehicle-treated animals did (Figure 9E,F).

## Discussion

Following axotomy, several metabolic changes occur in injured neurons to reinnervate denervated targets and thus ensure survival and regeneration. Moreover, a growth cone is formed at the tip of growing axons, and this process requires morphological and biochemical changes in the plasma membrane, including membrane extension and remodeling. In this context, cholesterol-rich lipid rafts, also called membrane microdomains, play a key role as signaling platforms of both growth-promoting and growth-inhibiting molecules. With regard to the possible contribution of lipid rafts and cholesterol to nerve regeneration, conflicting views can be found in the literature. Some authors demonstrated that lipid rafts promote axonal regeneration (Santuccione et al., 2005; Zhang et al., 2013) while others claim that they have an inhibitory role (Vinson et al., 2003; Hausott et al., 2011). These contradictions can be explained by the fact that, in addition to a variety of proteins and molecules that promote axonal regeneration, such as receptors of neurotrophic factors, lipid rafts also contain proteins and molecules that exert an inhibitory role, such as receptors of myelin-associated proteins (Kappagantula et al., 2014). On the other hand, there is also some discrepancy about the role of lipid rafts in cell apoptosis. It is described that pro-apoptotic receptors like Neogenin, FAS and its ligand, JNK, PKC, Src kinases and a wide variety of lymphocyte-related receptors are more functional when are localized in lipid rafts, inducing a line of investigation in which lipid rafts are targets for chemotherapy (George and Wu, 2012). However, receptors related with cell survival, like Akt cascade, also depend on lipid rafts (George and Wu, 2012). It has been shown that cholesterol depletion with MβCD protects cerebellar neurons from apoptosis (Zhou et al., 2012) and after spinal cord injury, lipid raft disruption promotes cell survival due to a loss of function of Neogenin (Tassew et al., 2014). In conclusion, some of these studies were performed *in vitro* and sought to address the cellular pathway or the signaling of a specific neurotrophin through lipid rafts, and did not take into account all the trophic factors and cell responses that occur *in vivo*, but instead focused on single pathways.

In the present study, we show that cholesterol depletion in juvenile cultured neurons increases the size of growth cones and enhances neurite and axonal extension and the marked formation of filopodium-like extensions in both central and peripheral neurons. Although we did not address the underlying molecular mechanisms, we believe that both the membrane fluidity induced by cholesterol depletion and partial lipid raft disruption may account for the effects observed, as extracellular matrix and adhesion receptors are also grouped in lipid rafts (Leitinger and Hogg, 2002; Decker and ffrench-Constant, 2004; Head et al., 2014). In addition, the increased size of neurites suggests that cholesterol depletion might be involved in the positive regulation of the exocytotic machinery needed for neurite growth (Ros et al., 2015).

Next, we addressed whether cholesterol depletion increases the regrowth of axotomized neurons in two *in vitro* models: dissociated hippocampal cultures and axotomized EC-hippocampal slice cultures. In both cases, we found a marked increase in the regenerative potential of lesioned axons, again suggesting that cholesterol depletion not only increases axonal length in developing axons but also axonal regrowth and regeneration *in vitro*. In addition, the observation that regenerated EC-hippocampal axons were correctly targeted to the appropriate termination layers (SLM and ML) indicates that cholesterol depletion does not alter the molecular mechanisms involved in the guidance and targeting of these axons to the hippocampus.

Membrane extension during axon growth after axotomy requires the incorporation of new lipids to the tips of the regenerating axons. Incorporated cholesterol in the axons of peripheral neurons after axotomy comes from transported cholesterol synthesized in the cell bodies of lesioned neurons (de Chaves et al., 1997), and from recycled cholesterol from cellular and myelin debris originated during the injury (Goodrum, 1991; de Chaves et al., 1997). The existence of multiple complementary mechanism to incorporate cholesterol into the re-growing membranes suggest a redundancy in the process of cholesterol reutilization. Our results *in vitro* and *in vivo* suggest that acute and medium-term reduction of cholesterol levels have positive effects for axon elongation and regeneration after nerve injury. However, further studies are required to evaluate long-term effects of systemic cholesterol reduction. Secondary undesired effects associated with alteration of membrane cholesterol levels should also have to be considered. Previous reports have found that MβCD may alter crayfish neuromuscular junction functioning in cold conditions (14°C) but not at 21°C (Ormerod et al., 2012) and cholesterol depletion in *Caenorhabditis elegans* results in decreased motility (Merris et al., 2004). Moreover, reduction of membrane cholesterol alters ion channel activity in sensory peripheral neurons (Saghy et al., 2015), with direct consequences for their hypersensitivity (Pristera et al., 2012) and the development of neuropathic pain (Ferrari and Levine, 2015; Amsalem et al., 2018). As a consequence, electrophysiological tests, pain threshold tests and locomotion studies were performed on healthy animals to evaluate any possible effect derived from the systemic MβCD administration and the consequent membrane cholesterol depletion. Our results on healthy mice showed no significant differences between treated and control animals with reported values similar to those found in previous studies in the literature (Verdu et al., 1996; Bruna et al., 2010; Ale et al., 2016). Of particular interest, the algesimetry results showed no indication of hyperalgesia in intact mice, and the absence of autotomy in mice in the regeneration experiment also points out that there was no increased pain sensitivity after lipid raft disruption (Casals-Diaz et al., 2009). Therefore, although some uncontrolled effect cannot be dismissed, 1 month of systemic MβCD administration and lipid raft disruption did not alter normal nervous system function in mice.

We then studied the effect of cholesterol depletion on peripheral nerve regeneration. To this end, we chose an *in vivo* model of peripheral nerve injury to avoid focusing on a single pathway or molecule, thus gaining a complete view of nerve regeneration. Cholesterol depletion and lipid raft disruption were achieved by means of treatment with MβCD (Zidovetzki and Levitan, 2007), a cyclic oligosaccharide that has been used as an antineoplastic (Grosse et al., 1998) and also as a complexing agent in pharmacological applications (Upadhyay et al., 2006). The latter use of MβCD is attributed to its amphipathic properties, and molecules can be encapsulated inside the cavity of cyclodextrins. Thus, MβCD may act not only by extracting cholesterol from the plasma membrane and disrupting lipid rafts but also by catalyzing neurotrophic factors and favoring their uptake by neurons or glial cells that play a role in peripheral nerve regeneration. This could explain the increase in the number of GAP43-positive DRG neurons, an observation that indicates that, after MβCD treatment, there are more neurons able to regenerate or that have switched to a regenerative state sooner.

Our results indicate that lipid rafts are disrupted from the cell membrane after MβCD treatment, as shown by the loss of CTxB staining. CTxB has affinity for the GM1 ganglioside, a member of the complex ganglioside family, which also includes GD1 and GT1 (Schnaar et al., 2014), and it has been widely used as a marker of lipid raft integrity (Kalka et al., 2001). It has been reported that treatment with MβCD destabilizes the link between the axon and myelin as a result of the reduction of GD1 and GT1 in the membrane. These two gangliosides act as a myelin-associated glycoprotein receptors (Vyas et al., 2002) and, consequently, they mediate the inhibition of neurite outgrowth. With lipid raft disruption by MβCD, gangliosides are removed from the cell membrane and they cannot mediate the inhibition of neurite outgrowth. This situation may confer the axon with a greater capacity to regenerate. Thus, the increased motor and sensory reinnervation we observed after axotomy and treatment with MβCD is consistent with a reduction of GD1 and GT1 inhibition.

Moreover, other studies have reported that cholesterol removal affects various modes of endocytosis (Rodrigues et al., 2013). Endocytosis is a widely used mechanism for receptor internalization from the cell membrane, and it has been demonstrated that cholesterol and lipid rafts play key roles in clathrin-independent endocytic (Lamaze et al., 2001) and in clathrin-dependent endocytic mechanisms (Rodal et al., 1999). Thus, with lipid raft alteration, endocytosis might be impaired, thereby also remodeling the normal presence or removal of different receptors at the cell surface. With cholesterol depletion by MβCD, the endocytosis of neurotrophic factor receptors may be inhibited (Hausott et al., 2011). As the improvement in motor and sensory regeneration suggests, this inhibition would lead to increased cell receptor availability at cell surface, thus facilitating axon regeneration. On the other hand, MβCD treatment might interfere with myelin as it has a high lipid and cholesterol content. Indeed, deficient cholesterol biosynthesis in oligodendrocytes delays myelination (Saher et al., 2005) and the maximal brain cholesterol synthesis corresponds to the peak of myelination process (Korade and Kenworthy, 2008). However, our results indicate that MβCD treatment did not affect myelination of the regenerated nerve fibers in the adult mice. Nevertheless, a detailed histomorphometric quantification of the regenerated axons might be needed for a quantitative evaluation of the degree of remyelination after lipid raft disruption.

Finally, our results indicate that MβCD treatment accelerates nerve regeneration after complete nerve lesion. The positive effect was detected as faster and higher levels of reinnervation of distal targets, such as plantar muscles, which are the most discriminative for detecting differences in nerve regeneration rate. Whether these results would be more pronounced after a more severe nerve injury requires further attention.

The results of this study shed light on the role of lipid rafts in peripheral nerve regeneration, although more research is needed to elucidate the specific mechanisms affected by lipid raft disruption. As this study was performed from a functional perspective, a number of alterations may have been occurring. On the one hand, we may have accelerated regeneration due to an increase in myelin clearance and thus a decrease in myelin-associated inhibitory agents. On the other, the beneficial effect might be due to an alteration of membrane ganglioside ratio or because lipid raft disruption promotes an environment that is favorable for neuron regeneration.

In conclusion, we have found that lipid raft disruption increases axonal growth in young neurons *in vitro*, enhances axonal regeneration in two *in vitro* models, and promotes peripheral nerve regeneration. Our results support the notion that these membrane microdomains play an important role in the signaling responsible for determining neural growth during development, as well as for nerve degeneration and regeneration after injury. This could be important because many pathways and molecules are involved in the processes that occur after nerve injury, and the environment plays a key role in the success or failure of nerve regeneration and functional recovery. However, caution should be taken before the translationability of our results. Considering the slow rate of nerve regeneration, in humans with considerable nerve length, target reinnervation may need several months to year. The possible effects of long-term lipid raft disruption treatment pointed above need to be investigated in preclinical models before any potential clinical application.

## Author Contributions

CR-B, RM-M, and MH-L designed, conducted, and analyzed the *in vitro* experiments. NO, JV, and XN designed, conducted, and analyzed the *in vivo* experiments. CR-B, RM-M, MH-L, and NO wrote the first draft of the manuscript. MP performed the organotypic experiments. AM did percoll gradients to isolated EGL neurons. RM-M, JV, XN, and ES designed the experiments, analyzed the data, and wrote the final version manuscript.

## Conflict of Interest Statement

The authors declare that the research was conducted in the absence of any commercial or financial relationships that could be construed as a potential conflict of interest.

## Supplementary Material

FIGURE S1 Electrophysiological, locomotion and algesimetry tests performed after vehicle or MβCD treatment in uninjured mice. **(A–D)** Electrophysiological results in mice. Results of CMAP amplitude **(A,B)** and latency **(C,D)** recorded in TA and PL muscles at 7, 14, 21, and 28 days after beginning of treatment. **(E)** Plot of the SFI obtained in walking track test. **(F)** Algesimetry test results during the 4 weeks of treatment.
